# Automated detection and classification of cognitive distortions: enhancing mental health diagnosis in Spanish-speaking populations

**DOI:** 10.1192/j.eurpsy.2026.10415

**Published:** 2026-07-31

**Authors:** A. M. Zakreva-Prykolota, M. De Prisco, M. Primé-Tous, A. Mas-Musons, X. Segú, M. Sanabra, C. Valenzuela-Pascual, M. Korniyenko, V. Oliva, G. Fico, J. Raduà, E. Vieta, D. Hidalgo-Mazzei, C. Escolano, G. Anmella

**Affiliations:** 1 Polytechnic University of Catalonia (UPC); 2 Hospital Clínic of Barcelona, Bipolar and Depressive Disorders Unit, IDIBAPS, CIBERSAM, University of Barcelona, Barcelona, Spain; 3 Imaging of Mood- and Anxiety-Related Disorders (IMARD) group, IDIBAPS, Barcelona, Spain., Barcelona, Spain

## Abstract

**Introduction:**

Cognitive distortions are systematic patterns of biased thinking strongly associated with a wide range of mental health disorders. However, most research on their automatic detection has focused on English-language corpora and relied heavily on opaque, black-box models, limiting clinical interpretability and cross-linguistic generalization.

**Objectives:**

This study addresses a critical gap by developing and evaluating interpretable machine learning methods for detecting and classifying cognitive distortions in Spanish-language text.

**Methods:**

A synthetic corpus of 1,509 paired distorted and alternative thoughts was generated by clinical psychologists. Using this dataset, models were trained and evaluated across three tasks: (1) binary detection of distorted thoughts, (2) detection in linguistically complex cases, and (3) multi-class classification into ten distortion categories.

**Results:**

A Support Vector Machine with TF-IDF bigram features achieved strong performance in binary detection (F1 = 0.9287). For multi-class classification, a Logistic Regression model with part-of-speech features attained the highest accuracy (F1 = 0.8436). Extending the setup to 11 classes—including non-distorted thoughts—yielded comparable performance (F1 = 0.7782) but highlighted challenges in generalizing to the non-distorted category. Feature analyses revealed close alignment with clinical theory, identifying key lexical indicators such as absolutist adverbs and first-person language.

**Conclusions:**

These findings demonstrate that classic, interpretable NLP approaches can provide accurate and transparent insights into distorted cognition. The study establishes a robust baseline for Spanish-language psychological text analysis and contributes to the development of automated, clinically meaningful tools for mental health support.

**Disclosure of Interest:**

None Declared

